# 1141. Incidence of adolescent syncope and related injuries following vaccination and routine venipuncture

**DOI:** 10.1093/ofid/ofad500.982

**Published:** 2023-11-27

**Authors:** Holly C Groom, Neon Brooks, Matthew T Slaughter, Kathleen Mittendorf, Allison L Naleway

**Affiliations:** Kaiser Permanente Center for Health Research, Portland, Oregon; Kaiser Permanente Center for Health Research, Portland, Oregon; Kaiser Permanente Northwest Center for Health Research, Portland, Oregon; Vanderbilt-Ingram Cancer Center, Vancouver, Washington; Kaiser Permanente Center for Health Research, Portland, Oregon

## Abstract

**Background:**

Vaccination is associated with syncope in adolescents. However, incidence of vaccine-associated syncope and resulting injury, and how it compares to syncope incidence following other medical procedures, is not known. Here, we describe the incidence of syncope and syncope-related injury in adolescents following vaccination and routine venipuncture.

**Methods:**

We identified all Kaiser Permanente Northwest (KPNW) members ages 9 to 18 years with a vaccination or routine venipuncture and a same-day ICD diagnosis of syncope and collapse from 2013 through 2019. All cases were chart reviewed to establish chronology of events (vaccination, venipuncture, syncope, and injury, as applicable) and determine whether the event was attributed to vaccination or venipuncture. Incidence rates for vaccine- and venipuncture-associated syncope were calculated overall and by sex and age group. Incidence rates for vaccine-associated syncope were also calculated by vaccine type (Human Papillomavirus (HPV) vs. non-HPV).

**Results:**

Of 197,642 vaccination and 12,246 venipuncture events identified, 549 vaccination and 67 venipuncture events had same-day codes for syncope. Chart validation confirmed 59/549 (10.7%) and 20/67 (29.9%) as vaccine-associated and venipuncture-associated syncope, respectively. Overall rates of syncope were 2.99 per 10,000 vaccination events (95% CI: 2.27-3.85) and 16.33 per 10,000 venipuncture events (95% CI: 9.98-25.21), yielding an incidence rate ratio of 0.18 (95% CI: 0.11-0.31). The incidence of vaccine-associated syncope increased with each additional simultaneously administered vaccine, from 1.51 (95% CI: 0.93-2.30) following a single vaccine to 9.94 (95% CI: 6.43-14.67) following 3 or more vaccines. When stratified by the number of vaccines received, rates of syncope were similar following vaccination events with and without the HPV vaccine. Among those with syncope events, documented injury occurred in about 15% of both vaccine and venipuncture events.

**Figure:**
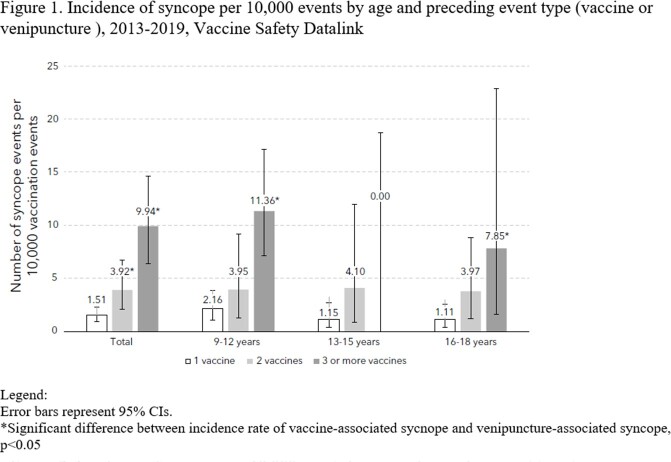

**Figure:**
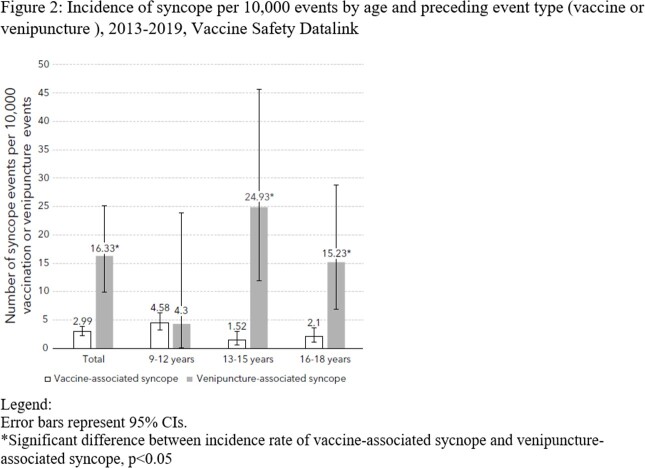

**Conclusion:**

Syncope occurs more commonly following venipuncture than vaccination. A higher number of simultaneously administered vaccines is a risk factor for post-vaccination syncope in adolescents.

**Disclosures:**

**Kathleen Mittendorf, PhD**, GE Healthcare: Grant/Research Support

